# Supplementary material for: Cluster analysis of transcriptomic datasets to identify endotypes of idiopathic pulmonary fibrosis
Source: Thorax. 2022 May 9;78(6):551–8. doi: 10.1136/thoraxjnl-2021-218563 (PMC9643664; doi:10.1136/thoraxjnl-2021-218563)
Supplement: Supplementary data [file thoraxjnl-2021-218563supp001.pdf]

Cluster analysis of transcriptomic datasets to identify endotypes of Idiopathic Pulmonary Fibrosis – online data supplement

Luke M. Kraven[1,2*], Adam R. Taylor[2*], Philip L. Molyneaux[3,4], Toby M. Maher[3,4,5], John E. McDonough[6], Marco Mura[7], Ivana V. Yang[8], David A. Schwartz[8], Yong Huang[9], Imre Noth[9], Shwu-Fan Ma[9], Astrid J. Yeo[2*], William A. Fahy[2*], R. Gisli Jenkins[3,4*], Louise V. Wain[1,10*]

Contents

Additional text 1
Systematic selection of publicly available datasets 1
Assignment of datasets to discovery and validation stages 2
Discovery stage studies 2
Validation stage studies 2
Data pre-processing 3
Data co-normalisation using COCONUT 3
Clustering using COMMUNAL 3
Comparison of phenotypic traits across clusters 4
Gene enrichment analysis 4
Developing the gene expression-based cluster classifier 5
Risk classification using the classifier 5
Risk classification using SAMS 5
Comparing prognostic methods using survival analysis 6
References 7
Additional Tables 8
Additional Figures 13

Additional text

Systematic selection of publicly available datasets

We performed our systematic search in March 2020 to select the datasets that were suitable for inclusion in the study (Figure E1). We required multiple sets of transcriptomic data from independent cohorts. We searched the Gene Expression Omnibus (GEO) (1) for all collections that contained the term 'IPF', excluding any that did not contain human samples. We restricted the search to collections with at least 30 samples as this allowed for inclusion of the largest datasets with the most IPF cases and healthy control subjects, which are the datasets that were the most likely to successfully co-normalise due to the higher counts of healthy control subjects. We did not restrict the search by platform. Each of the remaining collections were then reviewed to assess whether they contained data for IPF cases. All collections that did not contain data for IPF subjects were excluded.

For a successful co-normalisation and meaningful clustering results, we were required to choose an optimal tissue/cell type to use for the analysis. After reviewing the IPF datasets on GEO, we chose whole blood as our optimal tissue/cell type. There were three main reasons for this. Firstly, there were several relatively large whole blood datasets available on GEO and these would have provided the largest sample size and greatest statistical power for the study compared to other tissue types. Secondly, we required multiple datasets that contained data for healthy controls in addition to the IPF patients (so that the data could be co-normalised using COCONUT) and the whole blood datasets fulfilled this requirement. Thirdly, the accompanying clinical data for the whole blood datasets was far more comprehensive than for other tissue types, such as whole lung. This clinical data was vital

to the study as it was required for the characterisation of the clusters in both the discovery and validation stages. So, all GEO collections containing expression data measured from a non-blood tissue/cell type were excluded.

As multiple transcriptomic datasets were to be combined, it was important to check for the presence of common individuals across cohorts, which would have meant that the cohorts were not independent and could have biased the results of the study. To this end, the subjects in each collection were checked for unique study identification codes. Using these, we found that two of the blood collections, GSE132607 (n=74) and GSE85268 (n=68), both contained subjects from the Correlating Outcomes With Biochemical Markers to Estimate Time-progression in Idiopathic Pulmonary Fibrosis (COMET) study (ClinicalTrials.gov identifier: NCT01071707). There were a large number of IPF subjects in common between the two cohorts (n=58) and so we excluded the GSE85268 dataset as it was the collection with fewer IPF subjects.

The seven remaining collections of data were uploaded by research groups from across the USA (including the University of Virginia, Yale University, the University of Nevada and the University of Colorado) and the UK (Imperial College London). GSE27957 and GSE28042 were uploaded by the Kaminski Lab in Yale. These two collections were both used in the same study (2), where GSE27957 was used as discovery data and GSE28042 was used as independent replication data. Similarly, the data found in GSE133298 and GSE132607 were uploaded by researchers at the University of Virginia and were used as independent cohorts in the same study (unpublished as of October 2020, both collections uploaded to GEO in September 2019). All remaining collections were uploaded by separate research groups and no additional evidence of common subjects across cohorts was found so the seven cohorts of IPF subjects were deemed independent. However, the possibility that subjects could be common in two or more studies cannot be ruled out.

The human biological samples were sourced ethically and their research use was in accord with the terms of the informed consents under an institutional review board/ethical committee (IRB/EC)-approved protocol.

Assignment of datasets to discovery and validation stages

All cohorts included in the discovery stage must have contained healthy controls in order to enable the data co-normalization step. Four of the seven selected blood datasets contained data for healthy controls. We used the three with the greatest number of controls in discovery as these were the most likely to successfully co-normalize. The four remaining datasets were reserved for use in the validation stage. One dataset (GSE133298) was excluded during the validation stage as not all of the genes that were required to fully apply the classifier were present in the dataset.

Discovery stage studies

GSE38958: This dataset originates from an American observational study (3) that was investigating the relationship between sphingosine-1-phosphate lyase and pulmonary fibrosis. IPF cases were recruited from the University of Chicago. The authors studied gene expression data from peripheral blood mononuclear cells of IPF subjects (n=70) and compared this to gene expression from healthy controls (n=45).

GSE33566: This dataset contained data for 123 IPF subjects and 30 healthy controls. A subset of this data was used in an American observational study (4), where the authors hypothesised that a peripheral blood biomarker for IPF would be able to identify the disease in its early stages and allow for disease progression to be monitored. The IPF cases were recruited through the Interstitial Lung Disease or the Familial Pulmonary Fibrosis Programs conducted at National Jewish Health and Duke University. In the study, 40 IPF subjects were split into groups based on their predicted FVC and D_{LCO}, then the authors looked for differentially expressed genes between groups.

GSE93606: This dataset contained data from a British prospective cohort study (5) (n=57 IPF subjects and n=20 healthy age, sex and smoking history matched controls) which had the objective of examining host-microbial interactions in IPF subjects over time. IPF cases were prospectively recruited from the Interstitial Lung Disease Unit at the Royal Brompton Hospital, London, within six months of their initial diagnosis. The study was approved by the local research ethics committee (reference numbers 10/H0720/12 and 12/LO/1034). In this study, gene expression data from peripheral blood and lung function measurements were collected at multiple time points. However, only baseline gene expression and lung function data was used in our study. IPF patient survival was also recorded up to a maximum follow-up time of 34 months.

Validation stage studies

GSE132607: This dataset originates from a study (unpublished as of March 2022) which aimed to develop a predictor of FVC progression by studying gene expression differences in 74 IPF subjects over time. The subjects

included in this analysis were participants in COMET-IPF (Correlating Outcomes with biochemical Markers to Estimate Time-progression in Idiopathic Pulmonary Fibrosis), a prospective, observational study correlating biomarkers with disease progression. All IPF cases had been recruited in to this study within four years of their initial IPF diagnosis.

GSE27957 and **GSE28042**: both datasets originate from the same study (6), where the data in GSE27957 (n=45 IPF subjects) was used in discovery and the data in GSE28042 (n=75 IPF subjects) was used as independent validation data. Individuals with IPF from the GSE27957 dataset were recruited from the University of Chicago and the individuals with IPF from the GSE28042 dataset were recruited from the University of Pittsburgh. In brief, the authors used these cohorts to develop a 52-gene signature that had the ability to predict transplant-free survival in IPF subjects.

Data pre-processing

In each discovery dataset, probes that did not map to a gene were removed. In the instance where multiple probes mapped to the same gene, only the probe with the greatest mean expression was included in the analysis. Each dataset was then quantile normalised to reduce any technical differences between the gene probes within a study. Following this, each dataset was scaled so that all expression data was on the $\log_2$ scale and thus in a consistent form prior to co-normalisation. Genes were matched across studies based on their gene symbols.

Data co-normalisation using COCONUT

We used COmbat CO-Normalization Using conTrols (COCONUT) (7) (in R v4.0.0 and the 'COCONUT' package) to reduce the technical differences between the three discovery transcriptomic datasets, therefore enabling a cluster analysis to be performed on the pooled, co-normalized data. COCONUT is an unbiased co-normalisation method which assumes that all healthy controls across studies come from the same statistical distribution. It uses the healthy controls in each study to calculate correction factors that remove the technical differences in the data for the diseased subjects, without bias to the number of disease cases present. The method is adapted from the ComBat empiric Bayes normalization method (8), which is often used to adjust for batch effects within a study.

As COCONUT makes the assumption that all healthy controls come from the same background statistical distribution, we tested for significant differences in clinical and demographic traits between the healthy controls in each study, where possible. Clinical and demographic characteristics of the healthy controls were compared using chi-square tests for count data and analysis of variance for non-skewed continuous data.

Data for each study was input into COCONUT by providing a gene expression matrix (on the $\log_2$ scale) of common genes against subjects. These were accompanied by an indicator variable that showed which subjects were cases and which were controls. Following the co-normalisation, we removed all healthy control subjects from further analysis. Plots of the first two principal components of the transcriptomic data before and after COCONUT were used to evaluate the efficacy of the co-normalisation.

Clustering using COMMUNAL

In this study, we ran COMMUNAL using consensus clustering versions of two algorithms, K-means clustering and partitioning around medoids (PAM). Five different metrics were used to assess the validity of the clustering for different numbers of clusters and genes. These were: the gap statistic, connectivity, average silhouette width, the G3 metric, and Pearson's gamma coefficient. We ranked the genes in order of variance, with the 'top' 100 genes referring to the 100 genes with the greatest variance. We then applied the COMMUNAL algorithm using a range of input genes from the top 100 to the top 5,000. The genes with the greatest variance were used as these were the most likely to be informative, so as to minimise the number of non-informative genes and increase the signal-to-noise ratio.

The samples that were not assigned into the same cluster by the COMMUNAL clustering algorithms were labelled 'unclustered'. Since the intention was to use the clustered data to create a classifier and classifiers trained on data with fewer errors are more robust, these uncertain samples were removed from further analysis to improve the accuracy of the classifier.

The results were visualised in the form of a 3-dimensional (3D) map (Figure E2), which we used to select the optimal number of clusters in the data, as well as the optimal number of genes to use in the clustering. The map shows the mean of standardized values of each validity measure across the entire tested space. On the 3D map, blue squares indicate a potentially optimal clustering at a certain number of genes by finding the assignment where

Kraven LM, *et al. Thorax* 2023; 78:1–8. doi: 10.1136/thoraxjnl-2021-218563

the mean combined validation metric is greatest. The absolute maximum number of clusters for any consensus subset is marked with a red square. The points where the blue and red squares overlap indicate stable optima. If stable optima at a particular number of clusters are observed over most of the tested space, this indicates the presence of a strong, consistent biological signal at this number of clusters.

In Figure E2 there are stable optima at K=4 from 250 genes to 1,000 genes, and at K=3 from 2,500 genes to 5,000 genes, as shown by the red and blue squares meeting. Despite the K=4 clustering assignment at 1,000 genes showing the highest mean standardized validity score of all tested clustering assignments, there were stable optima at K=3 clusters over a larger range of tested space, indicating a stronger biological signal. As such, K=3 was chosen as the optimal number of clusters in the pooled IPF dataset. The clustering at 2,500 genes and 3 clusters was chosen as the optimal clustering assignment, under the assumption that the assignment with the fewest number of genes (out of those with stable optima at K=3) has the least amount of redundant signal.

Comparison of phenotypic traits across clusters

We characterised the clusters by comparing the clinical and demographic traits of the subjects that were assigned to each cluster. This was done for each phenotypic trait that was reported in at least one discovery cohort and one validation cohort. The statistical significance of the phenotypic differences across clusters was evaluated for all studies combined using a chi-square test for count data, an analysis of variance to compare means for non-skewed continuous data and a Kruskal-Wallis rank sum test to compare medians for skewed continuous data. For traits in the form of time-to-event data, Kaplan-Meier plots were used to approximate and visualise the survival function for these variables. Further, Cox proportional-hazards (PH) models were fit with cluster as the sole independent variable and the time to the event as the response variable.

Gene enrichment analysis

First, we assigned each of the 2,500 genes used in the optimal COMMUNAL clustering assignment to the cluster in which its expression was most different to its expression in the other two clusters, as this suggests that that gene was contributing to the identity of that cluster. 814 genes were assigned to Cluster 1, 866 were assigned to Cluster 2 and 820 were assigned to Cluster 3.

We then performed multiple ANOVA tests (one for each cluster) for each gene, each comparing the expression of that gene in subjects within a given cluster against the expression of subjects in both other clusters. Each gene was then assigned to the cluster in which it had the lowest ANOVA p-value. One benefit of this approach is that the ANOVA tests allowed for filtering based on statistical significance; a nominal p-value significance threshold of 0.05 was introduced and genes whose lowest ANOVA p-value was greater than this threshold were removed. The rationale for the introduction of this filtering step was that removing genes that were not associated with any cluster would reduce noise and strengthen the gene enrichment analysis for each cluster. The threshold for statistical significance was kept at a nominal level as a correction for all 7,500 ANOVA tests would have likely left too few genes assigned to each cluster to successfully perform the enrichment analysis. After the removal of the genes that were not at least nominally associated to any cluster, there were 769 genes assigned to Cluster 1, 839 assigned to Cluster 2 and 784 assigned to Cluster 3.

Then, gene enrichment analysis was performed separately on the three resulting gene lists using R v.4.0.0 and the in-house package ‘metabaser’ (database v20.3, package v4.2.3). This was used to search databases of gene ontology terms for statistically overrepresented *biological processes* and *biological pathways*. At the time that the analysis was performed, there were 17,552 *biological processes* and 12,222 *biological pathways* in the database accessed by metabaser. metabaser reports ‘q-values’, which are p-values that have been adjusted for multiple tests using the false-discovery rate. Gene ontology terms with q-value < 0.05 were deemed statistically significant. Sankey plots were used to show which of the genes that were assigned to each cluster corresponded to the 20 most significantly enriched *biological pathways* (see Figure 3).

Additionally, the gene lists of each cluster were searched for the presence of the nearest gene for any of the 14 variants that were genome-wide significant in Allen et al. (9), the largest genome-wide association study meta-analysis of IPF susceptibility to-date. The 14 genes were as follows: *AKAP13*, *ATP11A*, *DEPTOR*, *DPP9*, *DSP*, *FAM13A*, *LRRC34*, *IVD*, *KIF15*, *MAD1L1*, *MAPT*, *MUC5B*, *TERC* and *TERT*. Following this, enrichment analysis was performed on the genes of each cluster to investigate whether those genes were statistically overconnected (in terms of direct gene regulation) to any of the IPF-associated genes from Allen et al. (2020). If the genes that were assigned to a particular cluster were found to be overconnected to one or more of the IPF-associated genes listed above (say the exact number of overconnected IPF-associated genes is N), then a

hypergeometric test was performed to approximate the statistical significance of the finding that N out of the 14 IPF-associated genes were present within the list of overconnected genes for that cluster.

None of the 14 suspected IPF susceptibility genes from Allen et al. were assigned to Cluster 1, nor were they statistically overconnected to the genes that were assigned to this cluster. *FAM13A* was one of the genes that was assigned to Cluster 2, though it did not belong to any of the top 20 significantly enriched biological pathways. Additionally, the genes in Cluster 2 were statistically overconnected to five other IPF-associated genes. These were: *AKAP13*, *DSP*, *LRRC34*, *MAPT* and *TERT*. The hypergeometric p-value was calculated to be 0.020, indicating that it is significant that five IPF-associated genes were overconnected to the genes Cluster 2 and this is more than would be expected due to random chance. None of the IPF-associated genes from Allen et al. were found in the gene list for Cluster 3, although four were found to be statistically overconnected to the genes in this cluster. These were as follows: DSP, MAD1L1, MAPT and TERT. The statistical significance of this was approximated to be P=0.008 using a hypergeometric test, again indicating that this was significantly more than would be expected under random chance.

Developing the gene expression-based cluster classifier

Classification is a method of supervised machine learning that uses a correctly labelled training dataset to predict which category new observations belong in.

To determine the optimal genes to include in the classifier for the IPF data, we used an iterative algorithm which performed a greedy forward search for each cluster separately to determine the optimal combination of genes to differentiate between subjects in that cluster vs all other clusters. This was done by calculating receiver operating characteristic curves for each combination of genes and selecting the combination of genes which maximised the area under the curve (AUC). In an effort to prevent the classifier from being overfit to the discovery data, a threshold was implemented to stop the algorithm once an AUC of 0.99 had been reached. Each gene was labelled as either overexpressed or underexpressed based on whether the average expression of that gene was greater in the subjects from that particular cluster compared to the average expression across all subjects.

Making predictions with the classifier was a two-stage process. First, each subject was given a classification score for each cluster. This score was calculated as the geometric mean of the overexpressed genes for that cluster minus the geometric mean of the underexpressed genes. These scores were mean centred around zero and scaled to reflect a Z-score (i.e. standard deviation equal to 1). Ideally, subjects that belonged to a certain cluster should have had a high classification Z-score for that cluster and low classification Z-scores for the other clusters.

Then, we used the classification Z-scores to fit a multinomial logistic regression model, with cluster as the independent categorical variable and the Z-scores from each cluster as the dependent variables. This model had the ability to take data from new IPF subjects and predict which cluster they were each most likely to belong in, using only expression data from the optimal genes in the classifier. Importantly, the classifier does not use absolute levels of gene expression in order to make predictions, but instead utilizes relative gene expression between subjects. This meant that the classifier could be applied to a cohort of IPF cases (from the same study) without first requiring the removal of technical effects, which allowed for the use of validation datasets that did not contain data for healthy controls.

We tested the prediction accuracy of the classifier by using it to reassign all of the IPF subjects in the discovery datasets.

Risk classification using the classifier

Each of the IPF subjects in the two validation studies for which survival data was available, GSE27957 (n=45) and GSE28042 (n=75), were assigned into one of the three clusters using the 13 gene classifier. As significant differences in survival were observed between clusters 1 and 2 and 2 and 3, but not between clusters 1 and 3 (Table E9), we used assignment to clusters 1 and 3 to define high risk individuals and assignment to cluster 2 as low risk.

Risk classification using SAMS

Each of these individuals were also classed as high-risk or low-risk using SAMS (2). 7 of the 52 genes used by SAMS were expected to be more highly expressed in high risk cases than low risk cases (‘up genes’). Likewise, the remaining 45 genes were expected to be less highly expressed in high risk cases than low risk cases (‘down’ genes). The method that SAMS used to predict risk is as follows:

1. For each gene, the geometric mean of the expression for that gene across all subjects was calculated. This value represents the average level of expression for that gene across the whole cohort. It was then subtracted from the gene expression of that gene for each subject so that positive values represented subjects that had increased expression of that gene compared to the average and negative values represented subjects that had decreased expression compared to the average.

2. For each subject, the proportion of the 7 ‘up genes’ that were overexpressed was calculated. Similarly, the proportion of the 45 ‘down genes’ that were less highly expressed than average was calculated. So, if a subject had 4 ‘up genes’ that were greater than the average and 30 ‘down genes’ that were lower than the average, these proportions would have been 0.571 and 0.667 respectively.

3. For each subject, the sum of the geometric mean normalised expression data was summed up for the ‘up genes’ that were more highly expressed than average. Then the sum of the geometric mean normalised expression data was summed up for the ‘down genes’ that were less highly expressed than average. So, for example, for the subject above who had 4 of the 7 ‘up genes’ that were more highly expressed than the average, say with expression values 0.185, 0.553, 0.123 and 1.003 for these four genes, the sum would have been 1.864. The sum for the ‘down genes’ must always be negative, for example say that this sum for the subject above was -7.645.

4. The proportion of the ‘up genes’ calculated in step 2 was multiplied by the sum for the ‘up genes’ calculated in step 3 to produce the ‘up score’ for each subject. So, for the example subject above, their up score would have been $0.571\times1.864 = 1.064$. A ‘down score’ for each subject was also calculated by multiplying their proportion of down genes by their down sum from step 3. For our example subject, this would have been $0.667\times -7.645=-5.099$.

5. Subjects with up scores greater than the median value and down scores lower than the median value were classed as ‘high risk’, while all other subjects were classed as ‘low risk’.

This was done separately for each cohort and by using data from as many of the 52 genes as were measured in the datasets; 51/52 (98·1%) genes in the SAMS signature were present in GSE27957 and 50/52 (96·2%) were present in GSE28042. Two-way tables were used to compare agreement between the two methods.

Comparing prognostic methods using survival analysis

Kaplan-Meier plots were used to visualise the survival over time for the validation subjects in each risk group under each method. In both cases, the log-rank test was used to test the survival curves of each risk group for equality. Univariate Cox proportional-hazards models were fit to the data with risk group as the sole covariate and time-to-death as the outcome of interest. In both cases, the low-risk group was used as the reference group. The Concordance index (C-index), the equivalent of the area under the curve (AUC) for a receiver operating characteristic (ROC) curve, and the p-values from the log-rank test were used to assess which method performed best at assigning the IPF subjects to the correct risk group and therefore predicting survival.

Following this, multivariate Cox proportional-hazards models were used to assess whether the predictions made by each method were significant predictors of mortality in the validation datasets whilst adjusting for age, sex, ancestry, FVC and DL_{CO}. We used the likelihood ratio test and C-index to assess whether either of the two methods of risk prediction led to a significant increase in predictive ability over a Cox PH model containing only age, sex, ancestry, FVC and DL_{CO}.

Kraven LM, *et al. Thorax* 2023; 78:1–8. doi: 10.1136/thoraxjnl-2021-218563

References

(1) Edgar R, Domrachev M, Lash AE. Gene Expression Omnibus: NCBI gene expression and hybridization array data repository. *Nucleic Acids Res* 2002;30:207-210.

(2) Herazo-Maya JD, Sun J, Molyneaux PL, Li Q, Villalba JA, Tzouvelekis A, Lynn H, Juan-Guardela BM, Risquez C, Osorio JC. Validation of a 52-gene risk profile for outcome prediction in patients with idiopathic pulmonary fibrosis: an international, multicentre, cohort study. *The Lancet Respiratory Medicine* 2017;5:857-868.

(3) Huang LS, Berdyshev EV, Tran JT, Xie L, Chen J, Ebenezer DL, Mathew B, Gorshkova I, Zhang W, Reddy SP. Sphingosine-1-phosphate lyase is an endogenous suppressor of pulmonary fibrosis: role of S1P signalling and autophagy. *Thorax* 2015;70:1138-1148.

(4) Yang IV, Luna LG, Cotter J, Talbert J, Leach SM, Kidd R, Turner J, Kummer N, Kervitsky D, Brown KK. The peripheral blood transcriptome identifies the presence and extent of disease in idiopathic pulmonary fibrosis. *PloS one* 2012;7:e37708.

(5) Molyneaux PL, Willis-Owen SA, Cox MJ, James P, Cowman S, Loebinger M, Blanchard A, Edwards LM, Stock C, Daccord C. Host–microbial interactions in idiopathic pulmonary fibrosis. *American journal of respiratory and critical care medicine* 2017;195:1640-1650.

(6) Herazo-Maya JD, Noth I, Duncan SR, Kim S, Ma S, Tseng GC, Feingold E, Juan-Guardela BM, Richards TJ, Lussier Y. Peripheral blood mononuclear cell gene expression profiles predict poor outcome in idiopathic pulmonary fibrosis. *Science translational medicine* 2013;5:205ra136.

(7) Sweeney TE, Wong HR, Khatri P. Robust classification of bacterial and viral infections via integrated host gene expression diagnostics. *Science translational medicine* 2016;8:346ra91.

(8) Johnson WE, Li C, Rabinovic A. Adjusting batch effects in microarray expression data using empirical Bayes methods. *Biostatistics* 2007;8:118-127.

(9) Allen RJ, Guillen-Guio B, Oldham JM, Ma S, Dressen A, Paynton ML, Kraven LM, Obeidat M, Li X, Ng M. Genome-wide association study of susceptibility to idiopathic pulmonary fibrosis. *American journal of respiratory and critical care medicine* 2020;201:564-574.

BMJ Publishing Group Limited (BMJ) disclaims all liability and responsibility arising from any reliance placed on this supplemental material which has been supplied by the author(s)

Additional Tables

TABLE E1: Information about the transcriptomic data in the discovery datasets and the platform used in each study.

GEO accession	GSE38958	GSE33566	GSE93606
Microarray platform	Affymetrix Human Exon 1.0 ST Array	Agilent-014850 Whole Human Genome Microarray	Affymetrix Human Gene 1.1 ST Array
Number of gene probes	44,280	32,850	33,297
Number of unique genes	17,256	12,171	20,254

TABLE E2: Comparison of the age and sex of the healthy controls in each discovery stage study. Data are presented as count (percentage) or mean (standard deviation, SD). P-value for count data is from a chi-square test and the test comparing means is analysis of variance.

	GSE38958	GSE33566	GSE93606	P-value	n used
Number of healthy controls	45	30	20		
Age (years, SD)	69·3 (9·3)	62·4 (14·3)	66·0 (10·6)	0.187	83
Sex (% male)	27 (60·0%)	14 (46·7%)	12 (60·0%)	0.477	95

BMJ Publishing Group Limited (BMJ) disclaims all liability and responsibility arising from any reliance placed on this supplemental material which has been supplied by the author(s)

TABLE E3: Comparison of clinical and demographic traits of clustered discovery subjects by study and for all studies combined. Data are presented as count (percentage), mean (standard deviation, SD) or median (interquartile range, IQR). NA = data not available, FVC=Forced vital capacity, D_{LCO} = Diffusing capacity for carbon monoxide, FEV_1 = Forced expiratory volume in one second, CPI = composite physiologic index, MUC5B genotype = genotype for the MUC5B promoter polymorphism rs35705950. - indicates that the calculation was not applicable as there were zero subjects in that cluster. P-value for count data is from a chi-square test, test comparing means is analysis of variance and test comparing medians is the Kruskal-Wallis log rank test. Significant P-values ($P < 0{\cdot}05$) are highlighted in bold.

	**GSE38958 (n=65)**			**GSE33566 (n=83)**			**GSE93606 (n=48)**			**All studies combined (n=196)**				
	**Cluster 1**	**Cluster 2**	**Cluster 3**	**Cluster 1**	**Cluster 2**	**Cluster 3**	**Cluster 1**	**Cluster 2**	**Cluster 3**	**Cluster 1**	**Cluster 2**	**Cluster 3**	**P-value**	**Total n used**
n subjects in cluster	22	39	4	42	32	9	0	24	24	64	95	37		
**Age (years) (mean, SD)**	70·0 (6·3)	68·3 (7·9)	64·0 (2·7)	66·7 (9·8)	67·0 (14·1)	67·0 (12·1)	-	64·8 (5·9)	70·3 (8·8)	67·8 (8·9)	66·9 (10·2)	68·8 (9·4)	0·592	188
**Male (%)**	20 (91·0%)	30 (77·0%)	4 (100%)	32 (76·2%)	21 (65·6%)	3 (33·3%)	-	15 (62·5%)	16 (66·7%)	52 (81·3%)	66 (69·5%)	23 (62·2%)	0·091	196
**European ancestry (%)**	17 (81·0%)	29 (82·9%)	3 (75·0%)	NA	NA	NA	-	NA	NA	17 (81·0%)	29 (82·9%)	3 (75·0%)	0·883	60
**Ever smoker (%)**	NA	NA	NA	NA	NA	NA	-	15 (62·5%)	18 (78·3%)	NA	15 (62·5%)	18 (78·3%)	0·389	47
**Death observed during study (%)**	NA	NA	NA	NA	NA	NA	-	6 (25·0%)	16 (66·7%)	NA	6 (25·0%)	16 (66.7%)	**0·009**	48
**FVC % predicted (median, IQR)**	59·5 (19·5)	65·0 (24·0)	51·5 (7·8)	77·0 (36·0)	66·0 (46·0)	73·0 (17·5)	-	71·5 (27·7)	60·8 (24·1)	63.0 (35·0)	70·5 (30·1)	60·1 (23·4)	0·342	154
**D_{LCO} % predicted (median, IQR)**	34·5 (17·5)	49·0 (21·0)	28·5 (21·0)	65·0 (37·0)	66·0 (40·0)	30·0 (30·0)	-	38·1 (17·1)	36·6 (15·9)	35·0 (30·0)	45·0 (29·2)	34·4 (17·3)	**0·009**	133
**FEV_1 % predicted (median, IQR)**	NA	NA	NA	NA	NA	NA	-	74·9 (23·1)	65·4 (22·7)	NA	74·9 (23·1)	65·4 (22·7)	0·216	48
**GAP index (mean, SD)**	5·3 (1·3)	3·9 (1·3)	4·5 (1·3)	4·3 (1·5)	4·1 (1·6)	4·3 (3·1)	-	3·7 (1·8)	4·4 (1·6)	4·9 (1·4)	3·9 (1·5)	4·4 (1·7)	**0·006**	132
**MUC5B genotype: GG (%)**	NA	NA	NA	5 (29·4%)	6 (28·6%)	3 (60·0%)	-	5 (26·3%)	11 (50·0%)	5 (29·4%)	11 (27·5%)	14 (51·9%)	0·230	84
**MUC5B genotype: GT (%)**	NA	NA	NA	10 (58·8%)	14 (66·7%)	2 (40·0%)	-	12 (63·2%)	8 (36·4%)	10 (58·8%)	26 (65·0%)	10 (37·0%)		
**MUC5B genotype: TT (%)**	NA	NA	NA	2 (11·8%)	1 (4·8%)	0 (0%)	-	2 (10·5%)	3 (13·6%)	2 (11·8%)	3 (7·5%)	3 (11·1%)		

1

TABLE E4: The significantly enriched (q-value <0.05) biological processes for the 769 genes assigned to Cluster 1.

Biological process	Enrichment score	p-value	q-value
Mitochondrial ATP synthesis coupled electron transport	7.18	1.0×10^{-7}	7.8×10^{-4}
ATP synthesis coupled electron transport	7.12	1.2×10^{-7}	7.8×10^{-4}
Respiratory electron transport chain	6.88	1.4×10^{-7}	7.8×10^{-4}
Cellular respiration	5.95	1.3×10^{-6}	0.005
Oxidative phosphorylation	5.84	4.0×10^{-6}	0.012
Electron transport chain	5.56	4.3×10^{-6}	0.012
Homeostasis of number of cells	5.12	1.1×10^{-5}	0.024
Homeostatic process	4.54	1.7×10^{-5}	0.032

TABLE E5: The 20 most significantly enriched (q-value <0.05) biological processes for the 839 genes assigned to Cluster 2.

Biological process	Enrichment score	p-value	q-value
Cell activation	12.78	2.2×10^{-27}	3.7×10^{-24}
Immune system process	11.33	1.7×10^{-25}	1.4×10^{-21}
Leukocyte activation	11.76	2.4×10^{-23}	1.2×10^{-19}
Immune response	9.83	6.0×10^{-19}	2.5×10^{-15}
Regulation of immune system process	9.75	1.5×10^{-18}	4.9×10^{-15}
Regulated exocytosis	8.90	2.5×10^{-14}	6.9×10^{-11}
Response to stimulus	7.30	1.3×10^{-13}	3.1×10^{-10}
Defence response	8.16	1.6×10^{-13}	3.2×10^{-10}
Multi-organism process	7.74	1.9×10^{-13}	3.5×10^{-10}
Lymphocyte activation	8.73	4.5×10^{-13}	7.5×10^{-10}
Translational initiation	9.72	6.4×10^{-13}	9.1×10^{-10}
Symbiotic process	8.24	6.6×10^{-13}	9.1×10^{-10}
Interspecies interaction between organisms	8.02	1.6×10^{-12}	2.1×10^{-9}
Peptide metabolic process	8.31	1.9×10^{-12}	2.1×10^{-9}
Exocytosis	8.06	1.9×10^{-12}	2.1×10^{-9}
Peptide biosynthetic process	8.43	2.9×10^{-12}	2.9×10^{-9}
Translation	8.46	3.2×10^{-12}	3.1×10^{-9}
Regulation of biological quality	7.14	3.8×10^{-12}	3.5×10^{-9}
Myeloid leukocyte activation	8.09	4.1×10^{-12}	3.6×10^{-9}
Regulation of multicellular organismal process	7.20	5.0×10^{-12}	4.0×10^{-9}

TABLE E6: The 20 most significantly enriched (q-value <0.05) biological processes for the 784 genes assigned to Cluster 3.

Biological process	Enrichment score	p-value	q-value
Cell activation	20.78	1.3×10^{-60}	1.5×10^{-56}
Immune response	19.53	1.8×10^{-60}	1.5×10^{-56}
Leukocyte activation	20.87	3.3×10^{-59}	1.8×10^{-55}
Immune system process	18.04	1.6×10^{-57}	6.6×10^{-54}
Immune effector process	19.19	1.2×10^{-52}	4.0×10^{-49}
Myeloid leukocyte activation	20.63	1.7×10^{-52}	4.7×10^{-49}
Leukocyte activation involved in immune response	20.07	9.2×10^{-51}	2.2×10^{-47}
Cell activation involved in immune response	19.98	1.9×10^{-50}	3.9×10^{-47}
Neutrophil activation	20.19	1.0×10^{-48}	1.9×10^{-45}
Granulocyte activation	20.02	3.5×10^{-48}	5.7×10^{-45}
Neutrophil activation involved in immune response	19.55	4.0×10^{-46}	6.1×10^{-43}
Leukocyte degranulation	19.42	5.0×10^{-46}	6.8×10^{-43}
Neutrophil degranulation	19.43	1.3×10^{-45}	1.7×10^{-42}
Myeloid cell activation involved in immune response	19.21	1.5×10^{-45}	1.8×10^{-42}
Neutrophil mediated immunity	19.23	3.6×10^{-45}	3.9×10^{-42}
Myeloid leukocyte mediated immunity	18.99	1.1×10^{-44}	1.1×10^{-41}
Leukocyte mediated immunity	17.11	4.3×10^{-43}	4.2×10^{-40}
Secretion by cell	16.63	3.9×10^{-41}	3.5×10^{-38}
Export from cell	16.50	5.9×10^{-41}	5.2×10^{-38}
Defence response	15.95	1.2×10^{-40}	1.0×10^{-37}

TABLE E7: The 13 genes in the classifier. 'Up genes' refer to genes that were more highly expressed in the subjects for that cluster compared to the mean expression across all subjects, and 'down genes' refer to genes that were less highly expressed in the subjects in that cluster.

Cluster 1		Cluster 2		Cluster 3	
**Up genes**	**Down genes**	**Up genes**	**Down genes**	**Up genes**	**Down genes**
KCNK15	*RPF1*	*NOP58*		*CA4*	
SORBS1		*PSMA5*		*BCL2A1*	
HBB		*RASGRP1*		*UGCG*	
		IFI30			
		HLA-DRA			
		ATM			

TABLE E8: Coefficients of the multinomial logistic regression model fit using classification scores from the genes in the classifier. Note that Cluster 1 is the reference cluster and so the coefficients for this cluster are all zero and have been omitted.

Cluster	Intercept	Cluster 1 score	Cluster 2 score	Cluster 3 score
2	3.12	-9.75	8.87	1.66
3	-16.6	-11.92	-3.15	29.42

TABLE E9: Two-way tables comparing 'true' assignment of subjects from the discovery analysis (determined using COMMUNAL with 2,500 genes) to the reassignment of these subjects using the 13-gene cluster classifier.

		**True cluster**		
		Cluster 1	Cluster 2	Cluster 3
**Classifier predicted cluster**	Cluster 1	63	1	0
	Cluster 2	1	94	0
	Cluster3	0	0	37

TABLE E10: Pairwise comparisons showing the differences in survival over time between any two validation clusters, estimated using Cox proportional hazards models.

**Reference cluster**	**Alternate cluster**	**Hazard Ratio**	**95% CI**	**P-value**
Cluster 2	Cluster 1	3.80	1.78, 8.12	0.001
Cluster 2	Cluster 3	5.05	2.24, 11.35	9.1×10^{-5}
Cluster 1	Cluster 3	1.47	0.67, 3.22	0.341

TABLE E11: The agreement between the cluster classifier and SAMS when validation subjects were assigned to risk groups using each method.

**GSE27957 (n=45)**		Cluster classifier	
		High risk	Low risk
SAMS	High risk	13	2
	Low risk	5	25
**GSE28042 (n=75)**		Cluster classifier	
		High risk	Low risk
SAMS	High risk	17	12
	Low risk	19	27
**Both datasets combined (n=120)**		Cluster classifier	
		High risk	Low risk
SAMS	High risk	30	14
	Low risk	24	52

Kraven LM, *et al. Thorax* 2023; 78:1–8. doi: 10.1136/thoraxjnl-2021-218563

TABLE E12: Summary statistics from the Cox proportional hazards model adjusting for cluster, age, sex, ancestry, predicted forced vital capacity (FVC) and predicted diffusing capacity of the lung for carbon monoxide (DL_{CO}). OR = odds ratio, SE = standard error and CI = confidence interval.

Variable	OR	SE	P-value	95% CI
Cluster (high-risk cluster)	2.697	0.367	0.007	(1.315, 5.534)
Age (years)	1.006	0.020	0.748	(0.968, 1.046)
Sex (male)	5.720	0.752	0.020	(1.310, 24.969)
Ancestry (non-European)	1.099	0.608	0.876	(0.334, 3.619)
Predicted FVC	0.996	0.013	0.745	(0.971, 1.022)
Predicted DL_{CO}	0.967	0.013	0.008	(0.944, 0.991)

Additional Figures

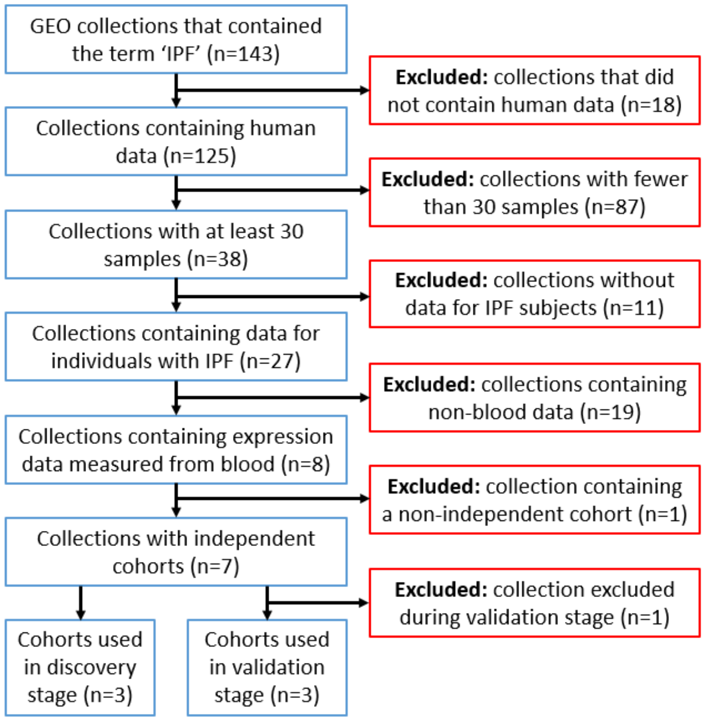

FIGURE E1: Flow diagram showing the process used to systematically select publicly available IPF gene expression datasets from the Gene Expression Omnibus for use in this study.

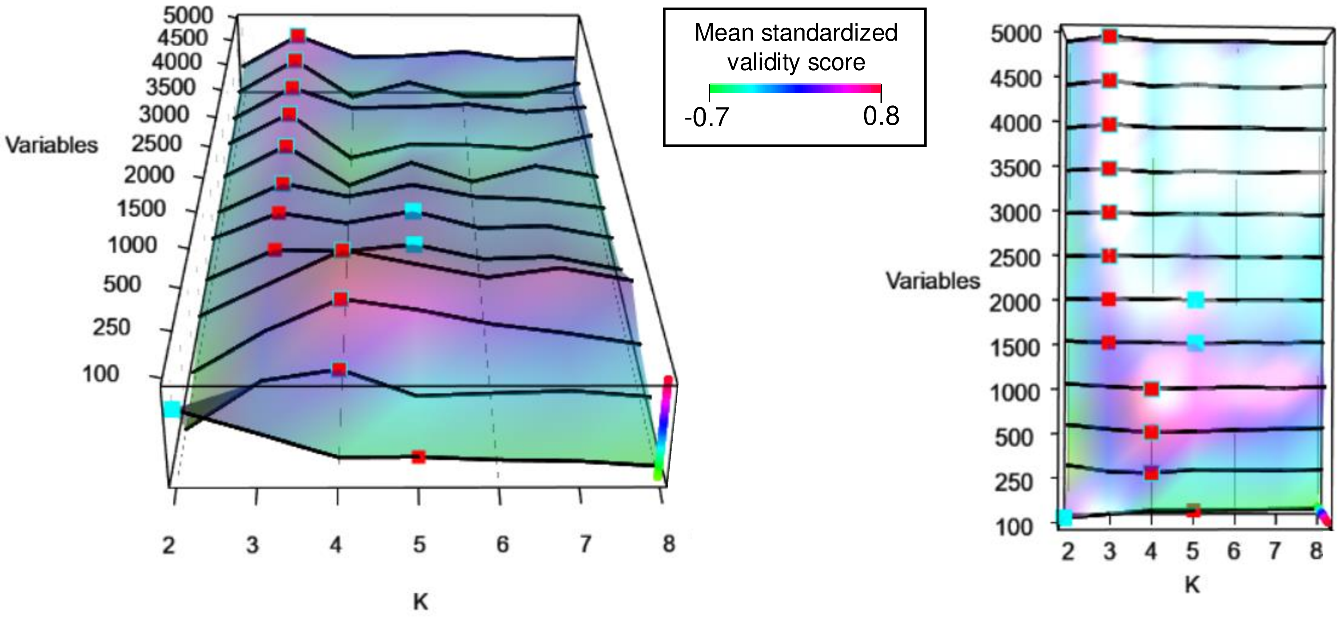

FIGURE E2: The 3D optimality map produced by COMMUNAL to identify the most robust number of clusters in the co-normalised data. A higher validity score indicates a better clustering assignment and stable optima are the points where the blue and red squares meet. In this map there are stable optima at K=4 from 250 genes to 1,000 genes, and at K=3 from 2,500 genes to 5,000 genes, as shown by the red and blue squares meeting. Despite the K=4 clustering assignment at 1,000 genes showing the highest mean standardized validity score of all tested clustering assignments, there were stable optima at K=3 clusters over a larger range of tested space, indicating a stronger biological signal. As such, K=3 was chosen as the optimal number of clusters in the pooled IPF dataset. The clustering at 2,500 genes and 3 clusters was chosen as the optimal clustering assignment, under the assumption that using the fewest number of genes has the least amount of redundant signal.

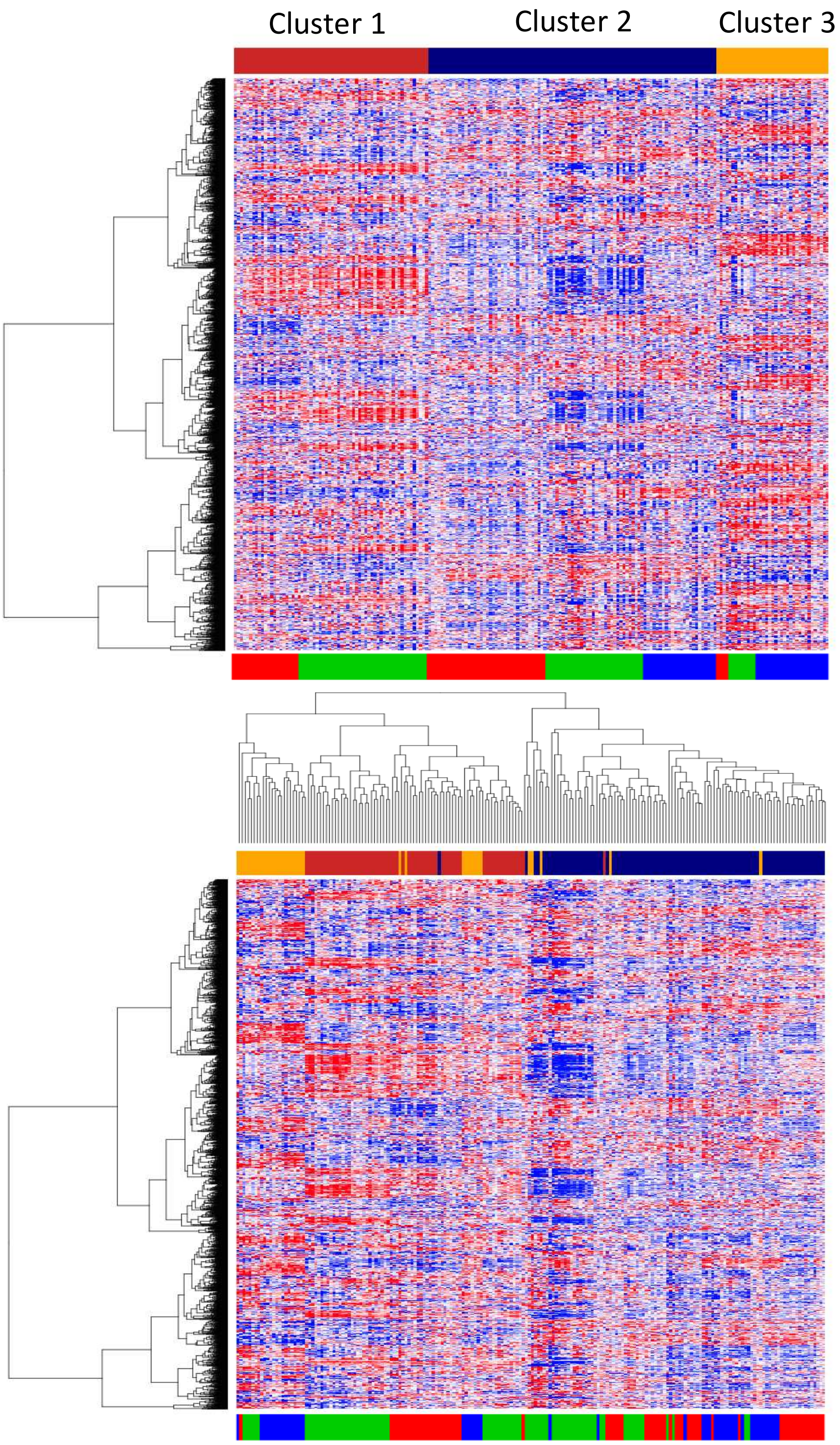

FIGURE E3: Heatmaps of gene expression for the clustered samples (x-axis) across the top 2,500 genes (y-axis), without hierarchical clustering of the samples (A) and with hierarchical clustering of the samples (B).
1 Blue inside the heatmap indicates low expression and red indicates high expression. In both plots, the genes have been hierarchically clustered for presentation purposes, the bar above the plot shows the cluster that subject was assigned in to (red = cluster 1, blue = cluster 2 and yellow = cluster 3) and the bar below the plot indicates which original study the subject was in (red = GSE38958, green = GSE33566 and blue = GSE93606).

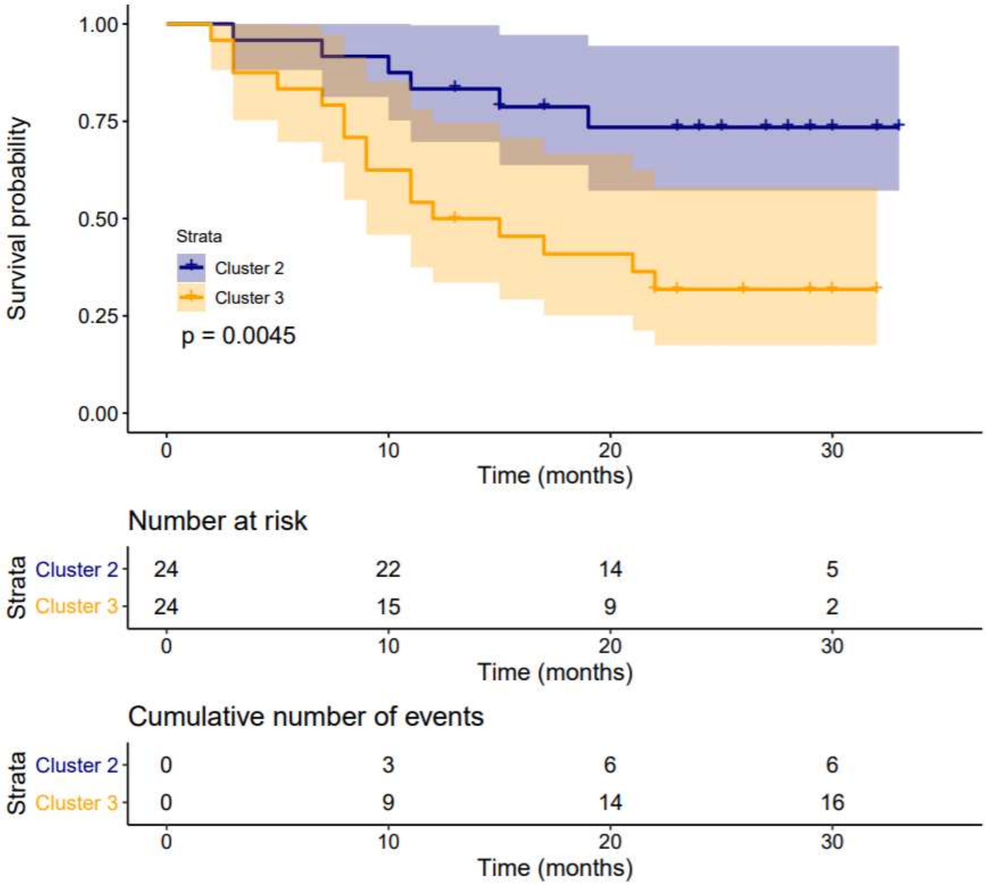

FIGURE E4: Kaplan-Meier curves and corresponding 95% confidence intervals showing survival over time for the subjects from study GSE93606, stratified by the cluster which they were assigned to in this study. The p-value shown on the plot is from a log-rank test testing the two curves for equality.

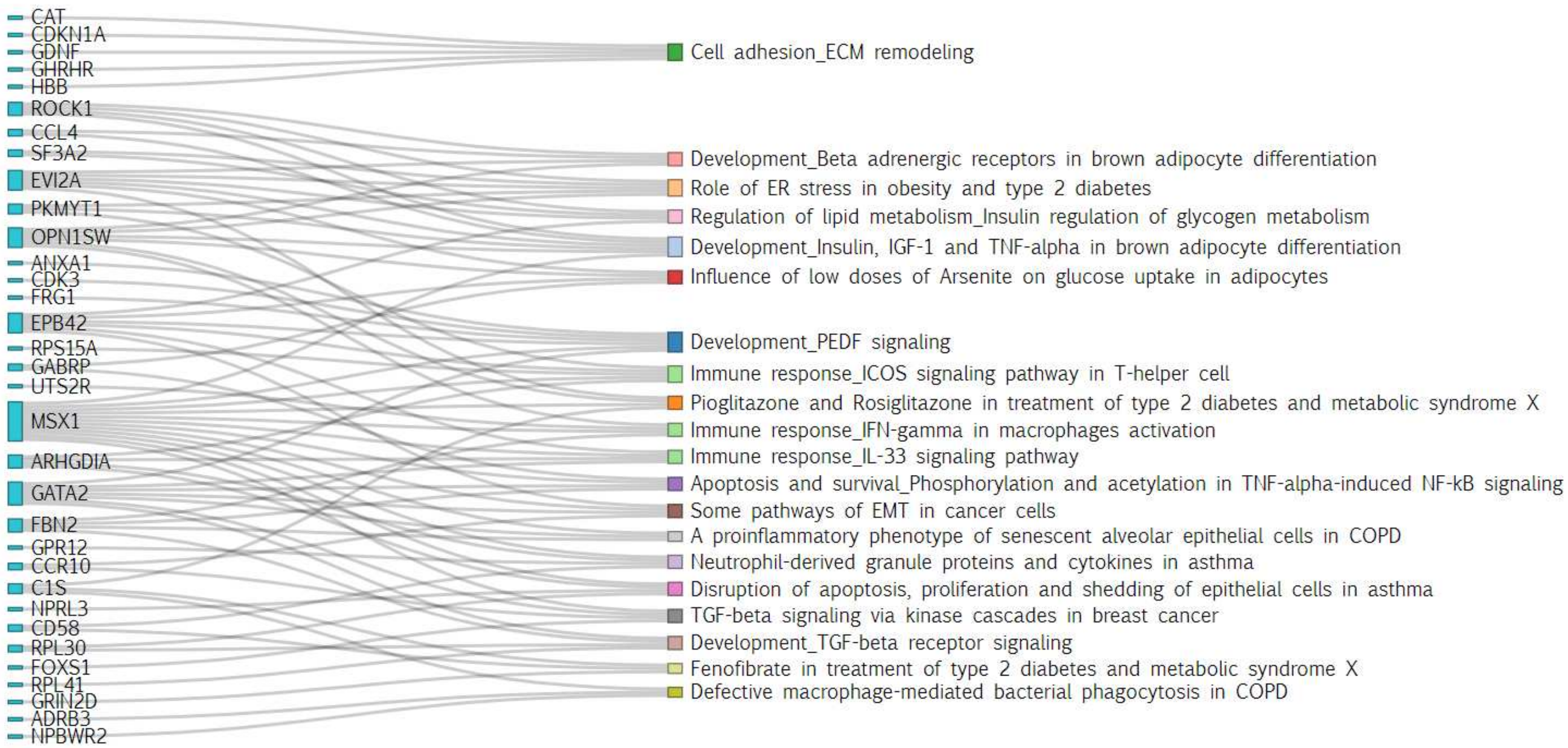

FIGURE E5: A Sankey diagram for Cluster 1 showing the genes that correspond to the 20 most significantly enriched biological pathways. The colour on the right hand side of the plot indicates the category of a particular pathway.

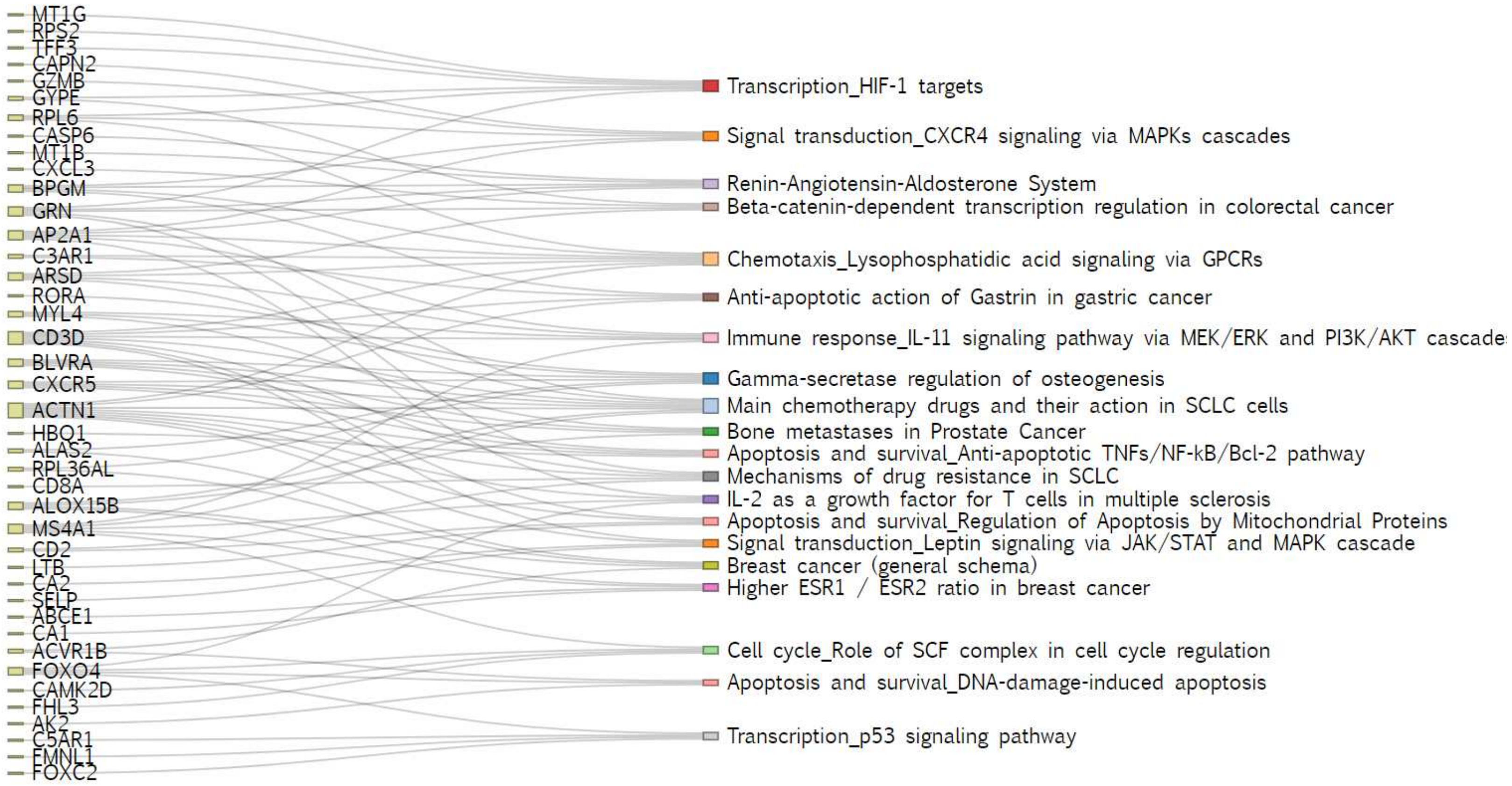

FIGURE E6: A Sankey diagram for Cluster 2 showing the genes that correspond to the 20 most significantly enriched biological pathways. The colour on the right hand side of the plot indicates the category of a particular pathway.

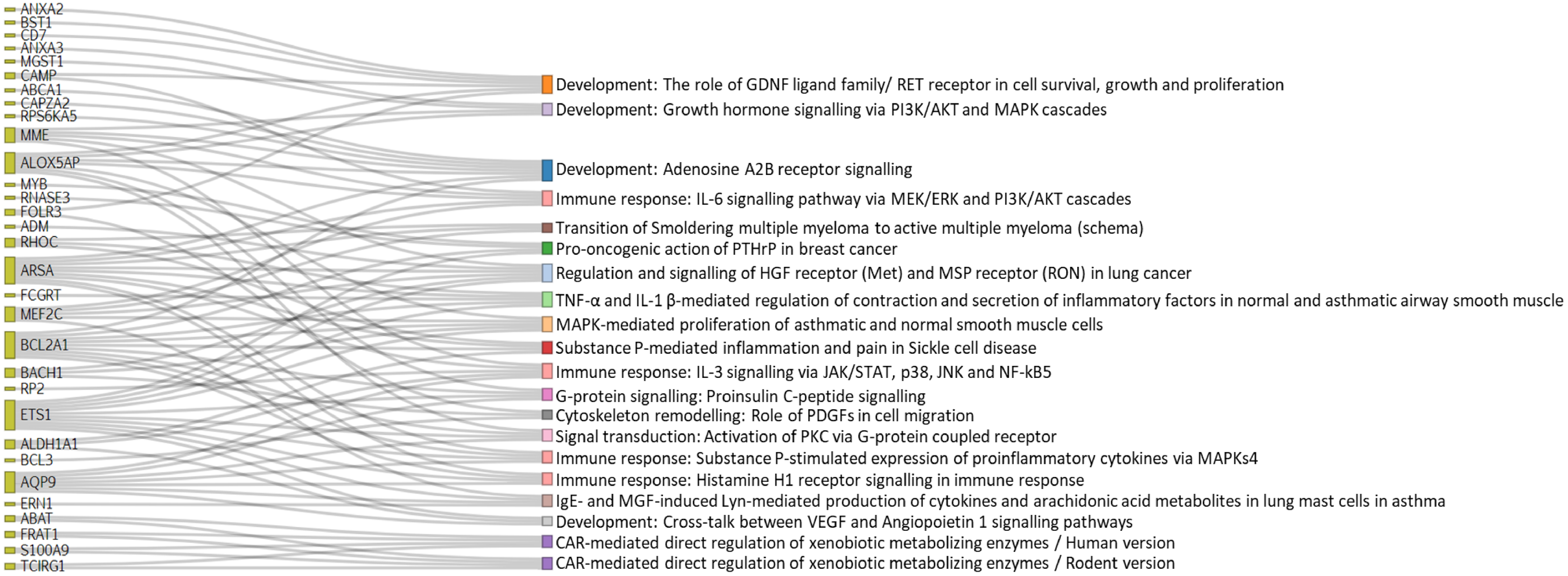

FIGURE E7: A Sankey diagram for Cluster 3 showing the genes that correspond to the 20 most significantly enriched biological pathways. The colour on the right hand side of the plot indicates the category of a particular pathway.

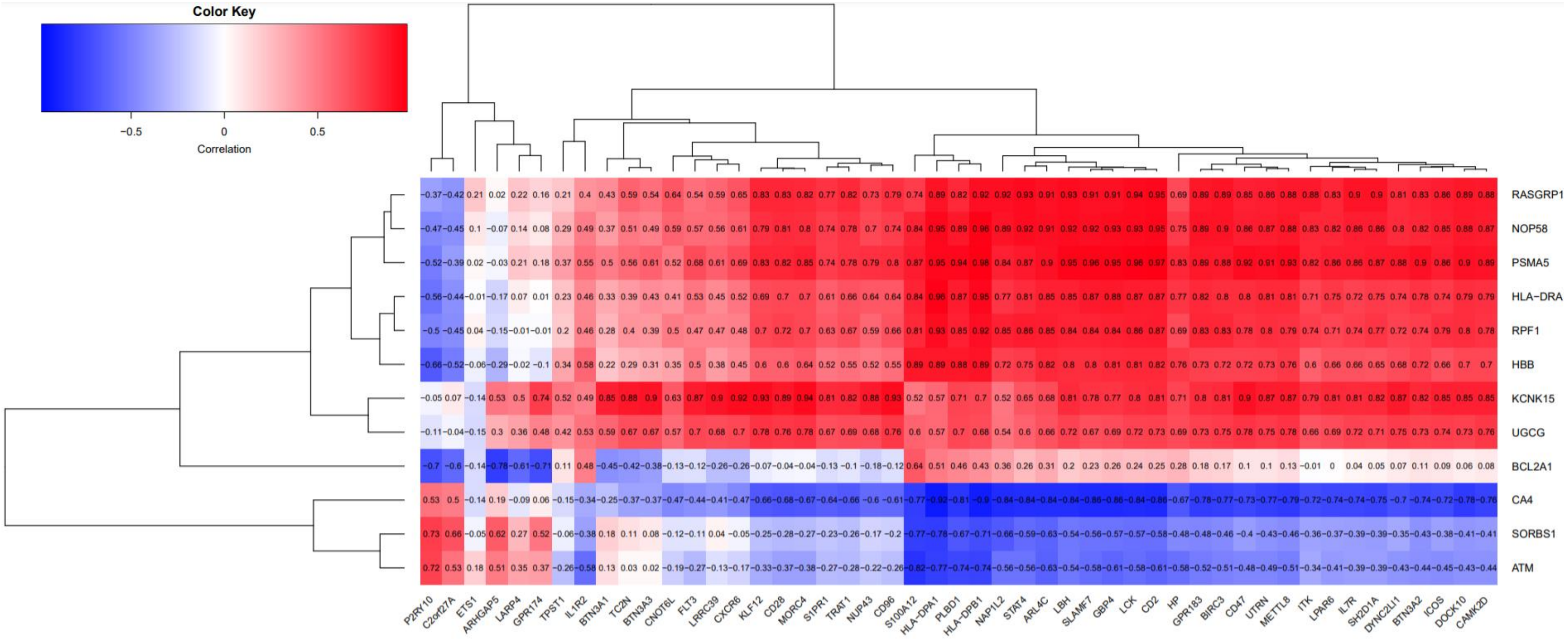

FIGURE E8: A heatmap showing the Pearson correlation between the genes in the classifier (y-axis) and the genes used by SAMS (x-axis). The correlation was calculated using the data from the IPF patients in the three validation cohorts (total n=194) for all genes that had complete data (12/13 genes for the classifier and 49/52 genes for SAMS). Both sets of genes were clustered using hierarchical clustering for presentation purposes.

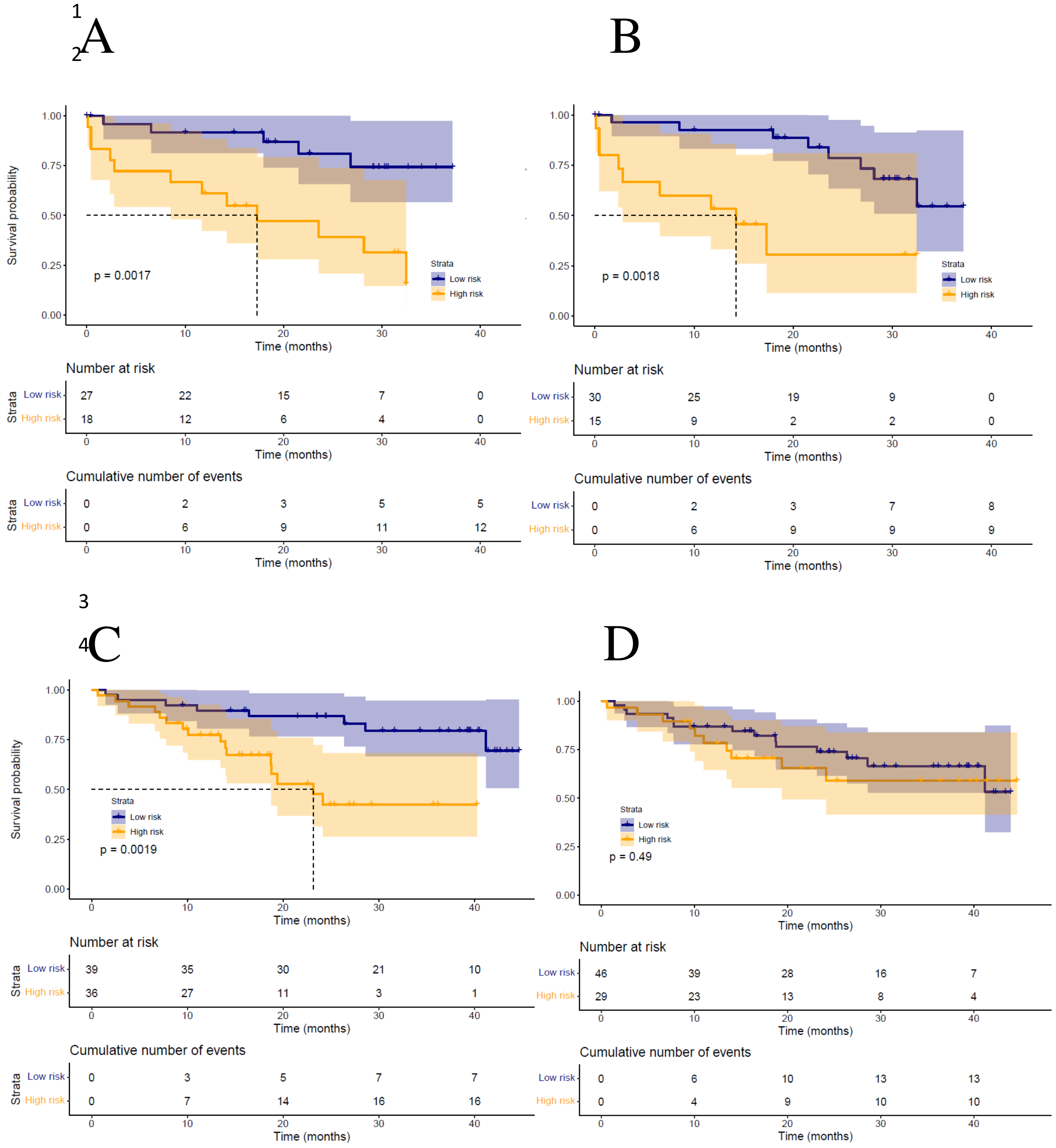

FIGURE E9: Survival over time for the IPF subjects in the validation datasets GSE27957 and GSE28042, stratified by predicted risk group. A) Survival of IPF cases from GSE27957 with risk predicted by our 13 gene classifier. B) Survival of IPF cases from GSE27957 with risk predicted by SAMS. C) Survival of IPF cases from GSE28042 with risk predicted by our 13 gene classifier. D) Survival of IPF cases from GSE28042 with risk predicted by SAMS. The P-value on each plot is from a log-rank test testing the two curves for equality. A dotted line on the plot indicates the median survival time for the risk group if this could be calculated.
